# Participant Attitudes Toward an Intensive Trial of Multiple Biopsies, Multidimensional Molecular Analysis, and Reporting of Results in Metastatic Triple-Negative Breast Cancer

**DOI:** 10.1200/PO.17.00076

**Published:** 2017-08-16

**Authors:** Nicole M. Kuderer, Kimberly A. Burton, Sibel Blau, Francis Senecal, Vijayakrishna K. Gadi, Stephanie Parker, Elisabeth Mahen, David Veenstra, Josh J. Carlson, Gary H. Lyman, C. Anthony Blau

**Affiliations:** **All authors**: University of Washington; **Vijayakrishna K. Gadi** and **Gary H. Lyman**, Fred Hutchinson Cancer Research Center, Seattle; and **Sibel Blau**, **Francis Senecal**, and **Stephanie Parker**, Northwest Medical Specialties, Puyallup and Tacoma, WA.

## Abstract

**Purpose:**

Multidimensional molecular analysis of tumor tissue intensively over space and time can provide insight into how cancers evolve and escape treatment. Attitudes of participants in such trials have not been assessed. We explored patient views regarding an intensive study incorporating multiple biopsies, multidimensional molecular testing, and drug response predictions that are reported to the oncologist and patient.

**Patients and Methods:**

A structured, self-administered survey was conducted among the first 15 patients enrolled in ITOMIC-001 (Intensive Trial of Omics in Cancer). Patients with metastatic triple-negative breast cancer were accrued at two sites in Washington state. Surveys containing 17 items were administered at enrollment and after the return of results. Surveys explored perceptions regarding risks, personal benefits, benefits to others, uncertainties associated with interpreting complex molecular results, concerns regarding multiple biopsies, and potential loss of confidentiality. At follow-up, three additional unique items explored patient coping.

**Results:**

All participants expressed a strong desire for their experiences to benefit others, and all perceived a higher likelihood of deriving benefit than described during detailed consent discussions. Loss of confidentiality ranked lowest among patient concerns. Despite acknowledging uncertainties and risks inherent in complex molecular testing for clinical reporting, participants wanted access to findings in evaluating treatment choices, even if the best available evidence was weak. Follow-up surveys demonstrated relatively little change in attitudes, although concern about study biopsies generally declined. Study participation helped several patients cope better with their disease.

**Conclusion:**

In advanced breast cancer, these findings demonstrate the feasibility of engaging motivated patients in trials that navigate the uncertainties associated with intensive spatial and longitudinal multidimensional molecular testing for the purpose of advancing precision medicine.

## INTRODUCTION

Molecular profiling can provide insight into tumor heterogeneity across space and over time,^1-10^ uncover patterns associated with treatment response and resistance,^11-17^ and ultimately, it is hoped, allow patients with cancer to receive drugs that are reliably effective.^18-24^ Because only a small fraction of the information extractable from a tumor sample can be exploited for therapy, many molecular drug-matching efforts rely on targeted sequencing.^25-29^ However, targeted sequencing fails to capture molecular features that may eventually prove clinically useful.^30-33^

The manner and extent to which results from complex molecular testing should be shared with patients remain subject to debate.^34-41^ Many oncologists are poorly equipped to interpret molecular test results^42-46^; no guidelines exist to inform these discussions,^39,43,45-47^ and patient preferences need to be better understood.^39,43,47-55^

Longitudinal monitoring can provide insight into how cancers evolve and escape treatment.^1,2,4,8-10,12-15,17,56,57^ However, repeated tumor biopsies are expensive and can cause serious complications.^58^ One method for circumventing this challenge relies on analyzing circulating cell-free DNA. However, we and others have found significant discordance between the results of next-generation sequencing testing of tumor tissue versus circulating cell-free DNA.^59-62^

To address these obstacles, we initiated ITOMIC-001 (Intensive Trial of Omics in Cancer; ClinicalTrials.gov identifier: NCT01957514). ITOMIC-001 enrolls patients with metastatic triple-negative breast cancer and involves repeated biopsies of multiple metastatic sites over time, an extensive multidimensional molecular analysis, a distributed network of investigators to analyze results and predict drug susceptibilities,^63^ and return of results to the patient and her oncologist. At their discretion, the patient can then be treated with the predicted drugs, allowing hypothesized drug susceptibilities to be tested in each patient.

ITOMIC-001 raises important questions regarding the engagement of patients with cancer in an intensive and exploratory research effort. Do patients with cancer understand the risks and uncertainties of receiving treatments on the basis of results from genome-wide analyses? What motivates their participation? How troublesome do patients find the prospect of undergoing multiple biopsies? Our objective was to address these and other questions using surveys administered to patients enrolled in ITOMIC-001.

## PATIENTS AND METHODS

### ITOMIC-001

#### Study design.

ITOMIC-001 was launched in October 2013 and has been described previously.^63^ For the first 15 patients described here, enrollment was restricted to those with metastatic triple-negative breast cancer who were platinum naive and scheduled to receive cisplatin. Research biopsies were performed before cisplatin administration, after discontinuation of cisplatin, and after discontinuation of subsequent therapies. Fifteen patients were enrolled by March 2016, and all surveys were completed by November 2016. ITOMIC-001 involves extensively characterizing the molecular features of a patient’s cancer with biopsies of up to seven metastatic sites (limited to two high-risk sites) in a single setting (metastatic sites were typically sampled between five and 10 times); performing whole-genome sequencing (or whole-exome sequencing), RNA sequencing, and deep sequencing of a panel of cancer-associated genes on multiple samples; employing a distributed network of experts to analyze findings and suggest treatments; reporting results to clinicians and participants for shared decision making; and repeating biopsies before switching to other drug treatments. Outside of the trial, we offer an intensive drug- and clinical trial–matching process to help patients who wish to be treated in accordance with our predictions access the recommended drugs. Patients were contacted 1 day and 1 week after biopsies to assess for adverse events (additional details provided in Data Supplement).

#### Informed consent.

The study has been approved by the Fred Hutchinson Cancer Research Center Institutional Review Board. The consent process was intended to clearly emphasize that patients are not expected to benefit directly from their participation, in addition to describing potential risks associated with extensive genomic testing (additional details provided in Data Supplement).

### Surveys

Participants were asked to complete structured, self-administered surveys that gauged their understanding and attitudes regarding key features of ITOMIC-001. Individual survey items and major thematic domains are listed in Table 1. Surveys were completed at baseline and within 1 month after receipt of the first molecular ITOMIC report describing expert panel findings. Surveys were completed at the patient’s convenience, typically at home, and research staff members were not present.

**Table 1.**
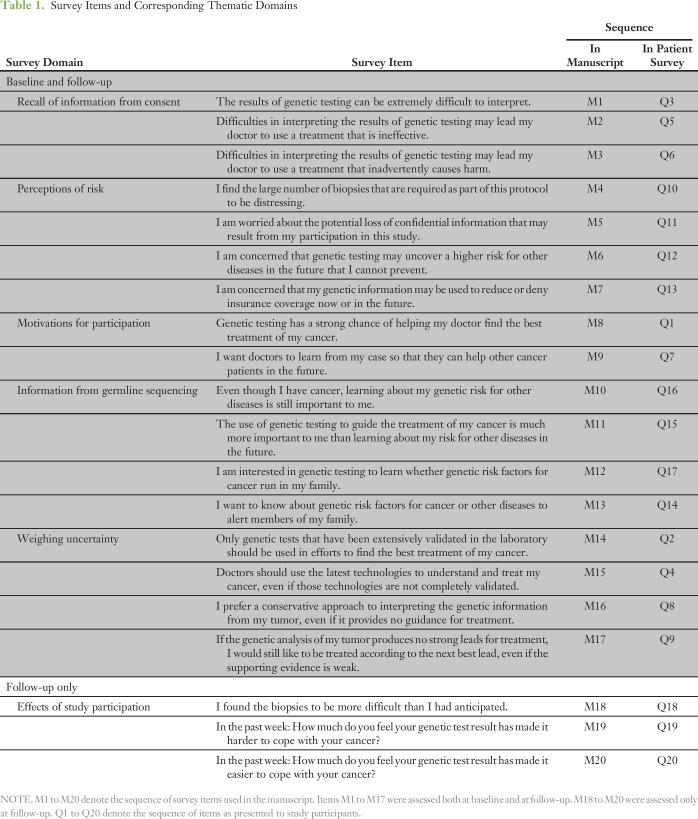
Survey Items and Corresponding Thematic Domains

### Statistical Methods

Survey questions were organized using a five-level Likert scale.^64^ Participants chose from among five responses for each survey item: agree strongly, agree a little, neither agree nor disagree, disagree a little, or disagree strongly. For purposes of analysis, these responses were transformed into five ordinal numeric values ranging from 2 (agree strongly) to −2 (disagree strongly). Descriptive statistical analyses of the numeric baseline and follow-up survey results are based on the mean and standard deviation (SD) calculated for each item across all respondents. Two of the three items unique to the follow-up survey varied response options because of the nature of the items (M19 and M20). These options were a great deal, a good deal, somewhat, a little, or not at all (Table 2).

**Table 2.**
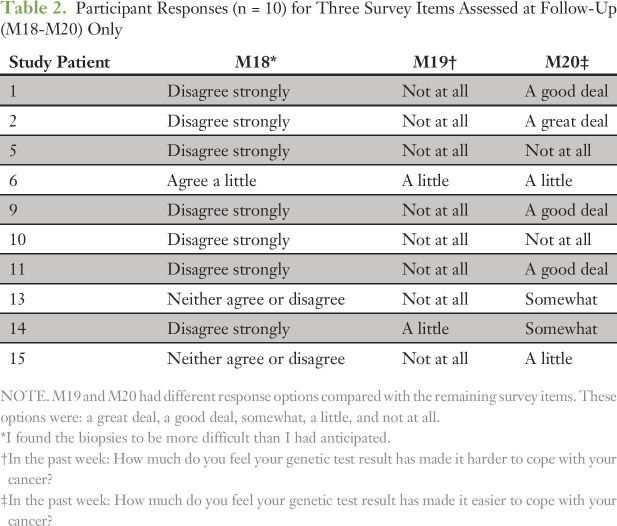
Participant Responses (n = 10) for Three Survey Items Assessed at Follow-Up (M18-M20) Only

## RESULTS

### Study Population and Survey Items

Survey items and major themes are listed in Table 1. Demographics of the 15 patients are summarized in Table 3 and the Data Supplement. Most participants were postmenopausal, employed, and married. Five of 15 participants did not complete the follow-up survey because of death (n = 3) or study withdrawal (n = 2). One participant required hospitalization for pain control because of skin biopsies that temporarily prevented her from taking the baths needed to alleviate discomfort associated with cutaneous infiltration of inflammatory breast cancer. No other study-related serious adverse events occurred. Among all participants, a total of 141 study-related biopsies were performed (mean per patient, 9.4; range, one to 18 biopsies), involving a total of 33 distinct anatomic sites (mean per patient, 2.2; range, one to five sites; Data Supplement). Ten patients (67%) were able to access a suggested drug or drug combination including approved drugs (n = 3) or off-label drugs (n = 9) through established clinical trials (n = 3) or single-patient investigational new drug applications (n = 3).

**Table 3.**
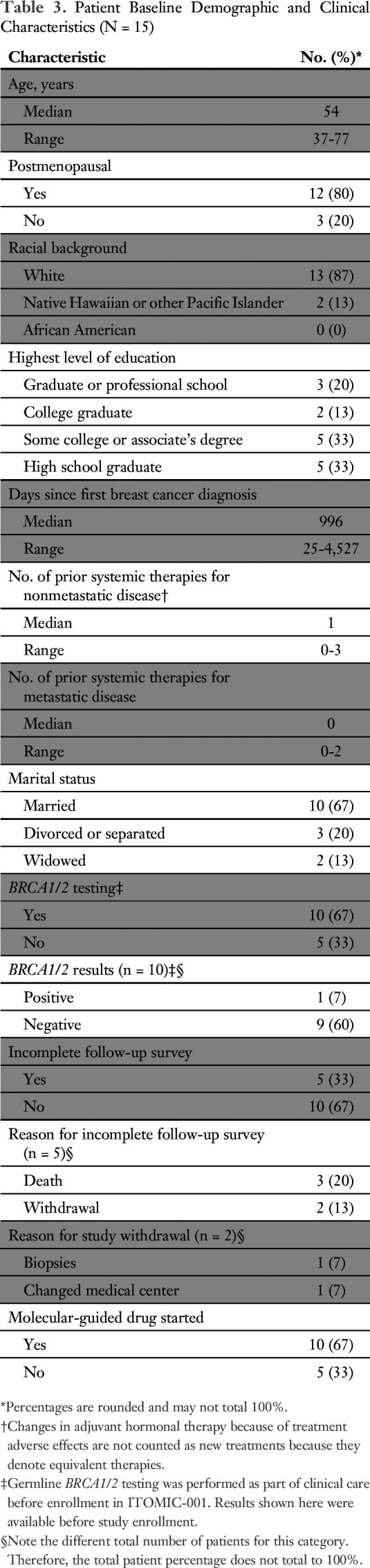
Patient Baseline Demographic and Clinical Characteristics (N = 15)

### Baseline Survey (M1-M17)

**Fig 1.**
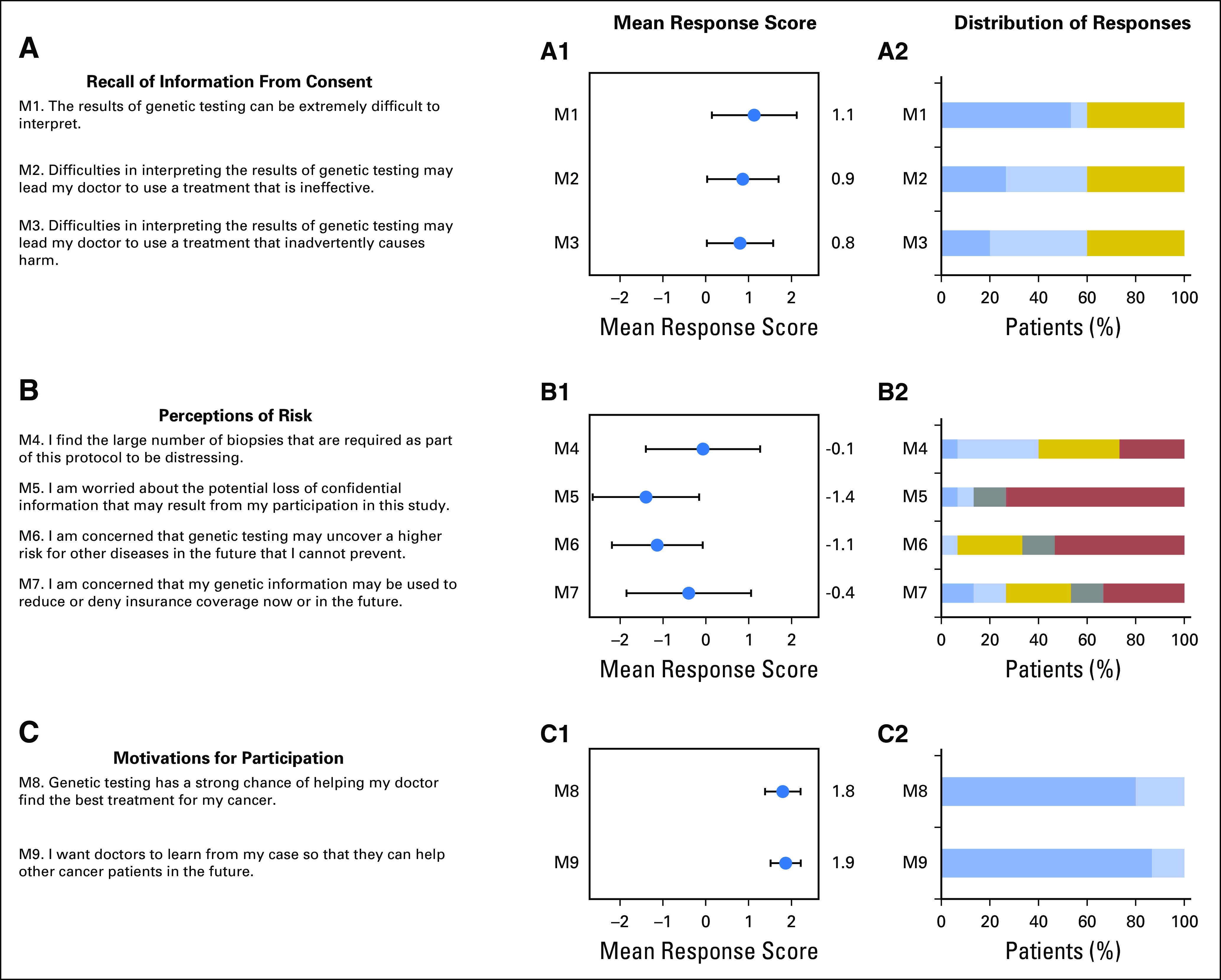

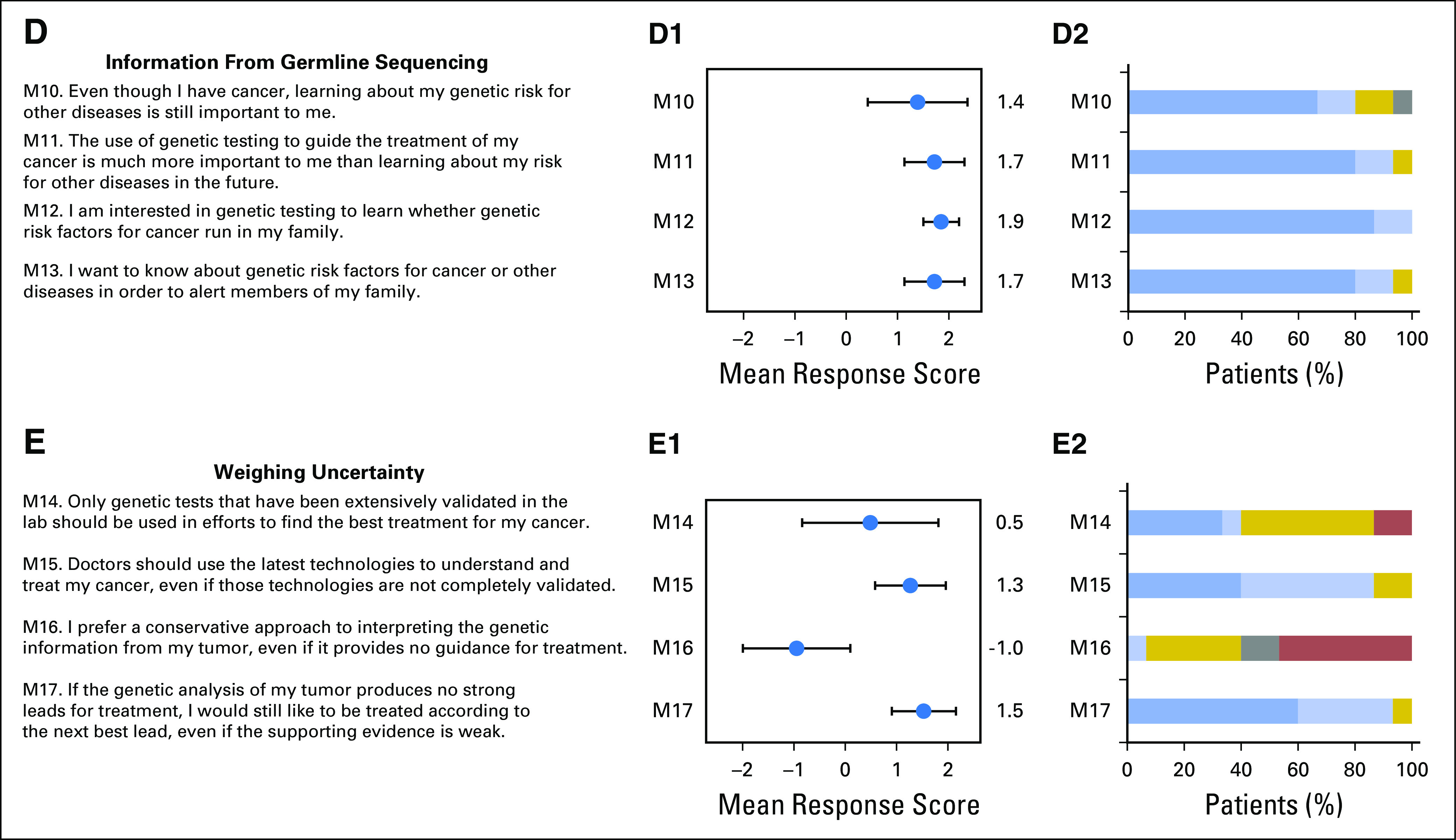
Mean response scores for each of the 17 survey items reported at baseline and distributions of individual responses (N = 15). (A-E) At the far left, each survey item is listed. (A1-E1) Left graphs display mean response scores on the *x*-axis, and whiskers denote standard deviations. The *y*-axis denotes individual survey items. Survey response scores correspond to: 2 = agree strongly, 1 = agree a little, 0 = neither agree nor disagree, −1 = disagree a little, and −2 = disagree strongly. (A2-E2) Right graphs show the number of patients in each response category; the *x*-axis reflects the percentage of patients in each category.Dark blue = agree strongly, light blue = agree a little, gold = neither agree nor disagree, gray = disagree a little, and red = disagree strongly.

Figure 1 depicts responses to the baseline survey items averaged across all 15 patients. Individual responses are shown in the Data Supplement.

#### Recall of information from consent (M1-3).

Three survey items assessed participants’ understanding of the complexities associated with multidimensional molecular testing. Responses were broadly consistent with information provided during consent (Fig 1A; Data Supplement). In response to survey item M1 stating “the results of genetic testing can be extremely difficult to interpret,” eight patients agreed strongly, one agreed a little, and the remaining six neither agreed nor disagreed. In response to M2 stating “difficulties in interpreting the results of genetic testing may lead my doctor to use a treatment that is ineffective,” four patients agreed strongly, five agreed a little, and six neither agreed nor disagreed. In response to M3 stating “difficulties in interpreting the results of genetic testing may lead my doctor to use a treatment that inadvertently causes harm,” three patients agreed strongly, six agreed a little, and six neither agreed nor disagreed. Whereas six patients responded neutrally to each item, only four provided neutral responses across all survey items in this category, and no patient disagreed with any of the items in this category. These results suggest that most patients understand the difficulties associated with interpreting results from high-dimensional molecular testing.

#### Perceptions of risk (M4-7).

Four survey items assessed concerns regarding risks associated with study participation (Fig 1B; Data Supplement). There was wide variation in responses to M4 stating “I find the large number of biopsies that are required as part of this protocol to be distressing.” One patient agreed strongly, five agreed a little, four disagreed strongly, and the remaining five neither agreed nor disagreed. In response to M5 stating “I am worried about the potential loss of confidential information that may result from my participation in this study,” one patient agreed strongly, one agreed a little, two disagreed a little, and 11 disagreed strongly. Regarding M6 stating “I am concerned that genetic testing may uncover a higher risk for other diseases in the future that I cannot prevent,” eight patients disagreed strongly, two disagreed a little, and only one agreed a little; there were four neutral responses. In response to M7 stating “I am concerned that my genetic information may be used to reduce or deny insurance coverage now or in the future,” five patients disagreed strongly, two disagreed a little, two agreed a little, and two agreed strongly; there were four neutral responses. These responses indicate wide variation among individuals in their perceptions of risks described in the consent. Participants tended to be most concerned about the biopsies associated with study participation and least concerned about a potential loss of confidentiality.

#### Motivations for participation (M8-9).

Two survey items focused on understanding motivations for study participation (Fig 1C; Data Supplement). All patients agreed strongly (n = 12) or a little (n = 3) with the statement that “genetic testing has a strong chance of helping my doctor find the best treatment for my cancer.” These responses contrasted with information provided during the consent process (Data Supplement). Virtually identical responses were obtained to the survey item stating “I want doctors to learn from my case so that they can help other cancer patients in the future,” with 13 patients agreeing strongly and two agreeing a little. These findings point to a near-uniform dual motivation for study participation: the potential for patients to obtain benefit themselves, which is significantly exaggerated compared with information provided at the time of consent, and the potential to benefit others.

#### Information from germline sequencing (M10-13).

All patients underwent either whole-exome sequencing (patients 1 to 12) or whole-genome sequencing (patients 13 to 15) of both germline (normal) and tumor DNA, and items M10 to M13 gauged perceptions regarding information from germline sequencing (Fig 1D; Data Supplement). Most patients agreed strongly (n = 10) or a little (n = 2) with item M10 stating “even though I have cancer, learning about my genetic risk for other diseases is still important to me” (one patient disagreed, and two were neutral). Almost all patients agreed strongly (n = 12) or a little (n = 2) with item M11 stating “the use of genetic testing to guide the treatment of my cancer is much more important to me than learning about my risk for other diseases in the future” (one patient gave a neutral response). Thirteen patients agreed strongly and two agreed a little with M12 stating “I am interested in genetic testing to learn whether genetic risk factors for cancer run in my family.” Twelve patients agreed strongly and two agreed a little with M13 stating “I want to know about genetic risk factors for cancer or other diseases in order to alert members of my family” (the one remaining patient was neutral). Collectively, all patients found some merit in germline testing.

#### Weighing uncertainty (M14-17).

Use of multidimensional molecular testing to infer drug susceptibilities for most cancers is in its infancy. Four survey items gauged patient views regarding if and how this information should be applied in their care (Fig 1E; Data Supplement). In response to M14 stating “only genetic tests that have been extensively validated in the lab should be used in efforts to find the best treatment for my cancer,” five patients agreed strongly, one agreed a little, seven neither agreed nor disagreed, and two disagreed strongly. In response to M15 stating “doctors should use the latest technologies to understand and treat my cancer, even if those technologies are not completely validated,” six patients agreed strongly, seven agreed a little, and two neither agreed nor disagreed. In response to M16 stating “I prefer a conservative approach to interpreting the genetic information from my tumor, even if it provides no guidance for treatment,” seven patients disagreed strongly, two disagreed a little, one agreed a little, and five neither agreed nor disagreed. Item M17 stated “if the genetic analysis of my tumor produces no strong leads for treatment, I would still like to be treated according to the next best lead, even if the supporting evidence is weak.” In response, nine patients agreed strongly, five agreed a little, and one remained neutral. Results suggest that patients want access to the latest technologies but have an expectation of diligence regarding the manner in which they are applied.

### Responses at Baseline Versus Follow-Up (M1-M17)

**Fig 2.**
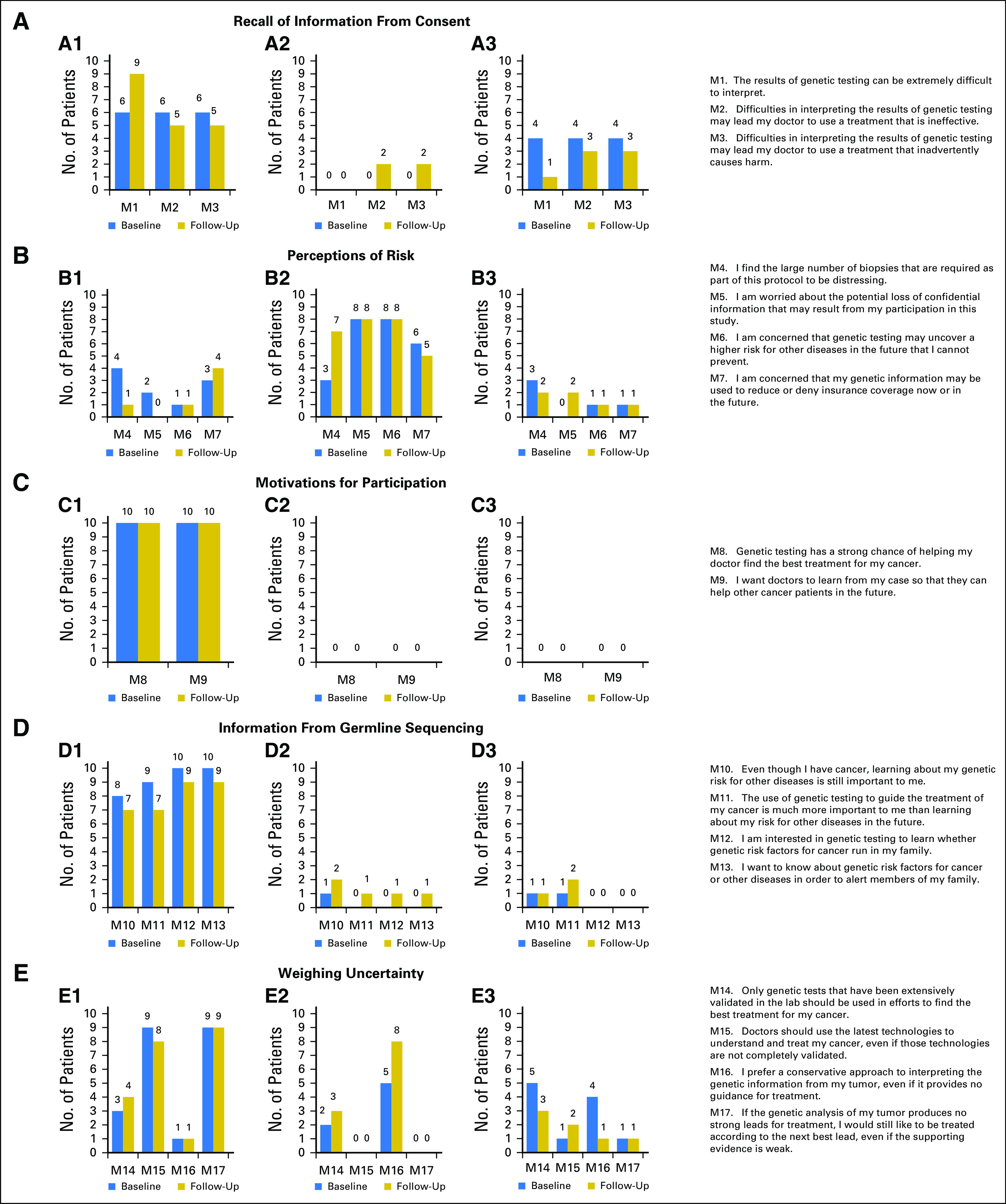
Comparison of responses at baseline and follow-up for the 10 patients who completed both surveys. (A-E) Survey item categories. (A1-E1) Left graphs indicate numbers of patients agreeing strongly or a little with the indicated survey item; (A2-E2) middle graphs indicate numbers of patients disagreeing strongly or a little with the indicated survey item; (A3-E3) right graphs indicate numbers of patients neither agreeing nor disagreeing with the indicated survey item. Numbers at the top of each column denote numbers of patients providing the indicated responses. Survey items M1 to M17 are listed on the right.

Figure 2 compares the responses of all 10 patients who provided both baseline and follow-up surveys. As shown in the Data Supplement, these 10 participants had nearly identical baseline responses compared with the overall study population. Although several participants changed their responses at follow-up (Data Supplement), the overall distribution of most responses remained remarkably stable (Fig 2). Most prominent were declines in concerns related to undergoing multiple biopsies (M4) and increasing rejection of a conservative approach to test interpretation (M16). Notably, motivations for study participation were unchanged at follow-up.

### Additional Items at Follow-Up (M18-M20)

Items M18 to M20 assessed how participation in ITOMIC-001 affected patient attitudes (Table 2). One patient “found the biopsies to be more difficult than anticipated” (M18), whereas seven of 10 disagreed strongly with this statement (two remained neutral). In response to M19 asking “in the past week: how much do you feel your genetic test result has made it harder to cope with your cancer,” two patients concurred a little, whereas the remaining eight concurred not at all. In response to M20 asking “in the past week: how much do you feel your genetic test result has made it easier to cope with your cancer,” two patients reported no improvement in coping, whereas the remaining eight reported at least some improvement (two a little, two somewhat, three a good deal, and one great deal).

## DISCUSSION

Linking comprehensive molecular profiling of distinct metastatic sites to treatments and responses holds promise for improving our understanding of how cancers evolve and escape therapy^1,3,4,9,10,17^ and for advancing personalized medicine.^6,45,46,56,57,65^ Here, we describe patient views regarding novel features inherent in ITOMIC-001, including concerns about multiple biopsies, weighing of uncertainties inherent in complex molecular testing, the value attached to germline testing, and motivations for study participation and its impact on patient coping. Follow-up surveys allowed us to assess changes in perceptions after having spent time in the study. Although not directly assessed in our surveys, patients enrolled in ITOMIC-001 also had a much higher likelihood (67%) of accessing the drugs that were predicted to be effective than patients enrolled in molecular drug-matching trials.^25,27,28,66,67^

The most consistent responses pertained to the motivations for participation. All participants expressed an exaggerated expectation of benefit, which contrasts information that had been provided during our detailed consent process. This previously described overestimation of personal benefit has been hypothesized to reflect expressions of hope and optimism rather than a misunderstanding of facts presented at the time of consent.^68-72^ Patients seemed to be similarly motivated by a desire that their experiences benefit others, consistent with recent studies suggesting that altruism can be an important motivator for participation in clinical trials.^73-75^

Ranking lowest among patient concerns were the potential for loss of confidentiality and the possibility that incidental findings from germline testing might uncover genetic risk factors for diseases other than cancer. Somewhat greater levels of concern were attached to the prospect of undergoing multiple biopsies and the possibility that information from genetic testing may be used to reduce or deny insurance coverage now or in the future. Encouragingly, concerns regarding multiple biopsies fell substantially in follow-up surveys. Nevertheless, one patient found the biopsies to be somewhat more difficult than expected at follow-up, and one of the two patients who dropped out cited concerns regarding multiple biopsies as the reason. ITOMIC-001 differs from commonly known mandatory research biopsy drug trials in that the final decision about drug matching is made by the treating oncologist outside the ITOMIC-001 protocol.

Most participants seemed to understand the uncertainties inherent in using complex molecular testing for making treatment recommendations. Responses regarding the manner in which results of complex molecular testing should be applied in reaching treatment decisions varied according to how the question was asked. Collectively, however, responses suggest that most patients understood the difficulties inherent in interpreting results from complex molecular testing but nevertheless wanted the opportunity to have these findings thoughtfully applied in their care.

All patients underwent germline testing, and all wanted access to results to assess their risk for genetic diseases other than cancer and to assess family members’ risk for cancer or other genetic diseases. However, 14 of 15 participants agreed with the statement that “the use of genetic testing to guide the treatment of my cancer is much more important to me than learning about my risk for other diseases in the future.” Incidental genomic findings can be perceived as burdensome,^48,52,54,55,76-78^ particularly in individuals with a low tolerance for ambiguity^54,55^ or in hypothetical scenarios.^79^ However, patients with advanced cancer making real-world decisions tend to perceive this information as either useful information for family^43,80^ or seemingly insignificant in the context of their cancer.^80^

Our study is limited by its small size and incomplete follow-up. Many of our patients were particularly motivated and may not be representative of the general cancer population. It is encouraging that eight of 10 patients viewed participation in ITOMIC-001 as having helped them cope with their cancer. Although this relatively high degree of satisfaction may be related to a high rate of accessing drugs that were predicted to be effective,^81^ both patients who felt that participation had made coping with their cancer a little more difficult had received treatments on the basis of molecular results. Notably, these same two individuals also indicated that genetic test results had made it easier to cope with their cancer, and it will be important to understand the basis for these responses to further improve satisfaction levels among all study participants.

Although preliminary, our findings establish the feasibility of partnering with motivated patients in intensive clinical trials that seek to better understand how cancers evolve and escape therapy. In exchange, patients expect best efforts in interpreting results (however difficult) and in accessing the drugs from which they hope to benefit.
